# Antidepressant medications have differential effects on REM sleep without atonia quantified by chin and upper extremity EMG

**DOI:** 10.1007/s44470-026-00127-2

**Published:** 2026-07-09

**Authors:** Jad El Ahdab, Carlos L. Rodriguez, Madeleine Grigg-Damberger, Matheus Lima Diniz Araujo, Noah D. Andrews, Sikawat Thanaviratananich, Nancy Foldvary-Schaefer

**Affiliations:** 1 Sleep Disorders Center, Department of Neurology, Neurological Institute, Cleveland Clinic, 9500 Euclid Avenue, S73, Cleveland, OH 44195 USA; 2 Department of Neurology, University of New Mexico, Albuquerque, NM USA

**Keywords:** REM sleep without atonia, REM sleep behavior disorder, Polysomnography, Antidepressants

## Abstract

**Objective:**

To determine associations between video polysomnography (vPSG)–quantified REM sleep without atonia (RSWA) and antidepressants (ADs) including selective serotonin reuptake inhibitors (SSRIs), serotonin norepinephrine reuptake inhibitors (SNRIs), and tricyclic antidepressants (TCAs).

**Methods:**

We analyzed 1474 vPSGs scored for possible REM sleep behavior disorder (RBD). RSWA scoring was based on submentalis and flexor digitorum superficialis (FDS) EMG using AASM criteria. Percent REM epochs meeting RSWA criteria (RSWA%) were compared by medication class in monotherapy or combination therapies using linear regression analysis, and adjusting for age, body mass index, gender, and race.

**Results:**

The sample included 1474 patients, 624 (42.3%) taking ADs and 850 (57.7%) non-users. Mean age was 53 ± 16.4 years, 53.1% male, and 73.1% White. Overall RSWA% was 11.9 ± 17.8. Compared to those medication-free, patients on SSRI or SNRI monotherapy had a 3.39% and 6.66% increase in RSWA%, respectively (*p* < 0.009 and *p* < 0.001); those on both SSRI and SNRI had a 10.44% increase (*p* = 0.089). In contrast, RSWA% did not increase in patients taking TCA monotherapy or the combination of SSRI + TCA or SNRI + TCA.

**Conclusion:**

Use of SSRIs and SNRIs, but not TCAs, is associated with increased RSWA% in a population with suspected parasomnia. The highest RSWA% estimates among combination groups were observed with SSRI + SNRI, although combination subgroup findings should be interpreted cautiously given small sample sizes. This study is the largest to quantify RSWA% and identify the differential impact of AD class, and monotherapy versus combination therapy on RSWA%. These findings should be interpreted within the context of the clinical criteria used for RSWA scoring.

**Supplementary Information:**

The online version contains supplementary material available at 10.1007/s44470-026-00127-2.

## Introduction

REM sleep without atonia (RSWA) is the polysomnographic (PSG) biomarker of REM sleep behavior disorder (RBD). RBD is a parasomnia characterized by loss of skeletal motor atonia normally present in REM sleep and dream enactment behaviors (DEB) [1]. Although isolated RBD in older adults is strongly associated with future development of α-synucleinopathies, including Parkinson’s disease (PD), dementia with Lewy bodies (DLB), and multiple system atrophy (MSA), increased REM sleep EMG activity is not specific to these disorders. RSWA may also be observed in association with antidepressant (AD) exposure, psychiatric disease, narcolepsy, and other neurologic or sleep disorders [2, 3]. The ICSD-3-TR requires RSWA on PSG to confirm the diagnosis of RBD.

Depression also precedes the onset of many neurodegenerative and sleep-related conditions, and ADs are often prescribed [4]. Symptoms of depression, anxiety, and AD use have been identified as potential risk factors for RBD. Furthermore, RSWA is more common in psychiatric populations than the general population and associated with AD use and symptoms of depression or anxiety [5, 6].

Selective serotonin reuptake inhibitors (SSRIs) and serotonin norepinephrine reuptake inhibitors (SNRIs) are reported to be associated with RSWA [7–10]. Lee et al. found RSWA on PSG in 176(12.2%) of 1444 adults on ADs, a tenfold higher prevalence compared to the entire sleep laboratory population. Only 7 (0.48%) had RBD [9]. Frauscher et al. recommend that RBD can be diagnosed on PSG if > 27% of REM sleep epochs contain RWSA using the Sleep Innsbruck Barcelona (SINBAR) montage (submentalis phasic/tonic + phasic right and left flexor digitorum superficialis (FDS)) [11].

Only a few small case–control studies have quantified the percentage of REM sleep epochs containing RSWA in relation with the use of AD using different methodologies. Winkelman et al. (2024) found RSWA in 9.54% ± 9.06 of all REM sleep epochs in 15 serotonergic AD users versus 2.36% ± 3.88 in controls (*p*= 0.02) recording submentalis and anterior tibialis (AT) electromyography (EMG) [10]. McCarter et al. reported mean RSWA% of 36.9% ± 21.3 in 30 AD users versus 15.9% ± 8.4 in 15 non-users (*p*< 0.01) [12]. However, both studies used older RSWA quantification methods, were limited by small sample sizes, and relied on AT EMG which is susceptible to artifact and false positivity [11].

Tricyclic antidepressants (TCAs) can also cause or unmask RSWA and RBD, although RSWA% has never been quantified [13]. While ADs have been associated with the emergence of secondary RBD that resolves with medication discontinuation [14, 15], newer evidence posits that ADs may unmask pre-existing RBD rather than cause it, serving as a potential early marker of subclinical neurodegeneration [16].

No study to date has quantified the magnitude of effect of ADs particularly by class on RSWA% using AASM scoring criteria and the SINBAR montage incorporating upper extremity EMG. Our goal was to compare RSWA% in patients on SSRIs, SNRIs, and/or TCAs vs. non-users in a large PSG database using FDS EMG.

## Methods

### Study design

This is a retrospective cross-sectional study using data from the Sleep Signals, Testing, And Reports Linked to Patient Traits (STARLIT) registry (the sleep laboratory database of the Sleep Disorders Center at Cleveland Clinic). We included vPSGs performed between September 2018 and October 2023. Medication use, demographic variables, and PSG-derived RSWA measures were collected retrospectively from the electronic health record (EHR) and STARLIT registry at the time of vPSG. The study was approved by the institutional review board.

### Sample size

RSWA was scored on 1474 vPSGs of adult patients, 624 taking SSRIs, SNRIs, or TCAs and 850 non-users. Included cases had at least 5 min of REM sleep recorded and at least one of the following: (1) suspicion of parasomnia by history as determined by the ordering provider; (2) a parasomnia type behavior observed on vPSG; (3) at least 10% of REM epochs meeting RSWA criteria by technologist visual review; (4) pre-sleep questionnaire positive for a parasomnia type symptom; or (5) patient report of parasomnia symptom during vPSG on the post-study questionnaire.

These inclusion criteria reflect the clinical nature of our sleep laboratory database. Manual RSWA scoring, which is highly labor intensive, was not performed for all patients undergoing PSG. Instead, RSWA scoring was performed clinically for vPSGs with possible parasomnia. Therefore, the inclusion criteria reflect the clinical workflow used to identify studies requiring RSWA scoring. This selection process enriches the cohort for patients who are more likely to have RSWA and should be considered when interpreting the absolute RSWA percentages reported in this study.

### Variables and data measurements

We collected SSRI, SNRI, and TCA use (Supplemental Table) and demographic data at the time of vPSG from the Cleveland Clinic EHR. PSG data were obtained from the Cleveland Clinic STARLIT registry.

Submentalis and left and right FDS EMG recordings were acquired and RSWA manually scored in accordance with The AASM Manual for the Scoring of Sleep and Associated Events Version 2.6 [17, 18]. Electrode placement, impedance, and signal quality were assessed by polysomnographers to ensure optimal RSWA quantification. To prevent confounding by sleep disordered breathing, REM epochs during or within 30 s of a respiratory event were excluded.

**Fig. 1:**
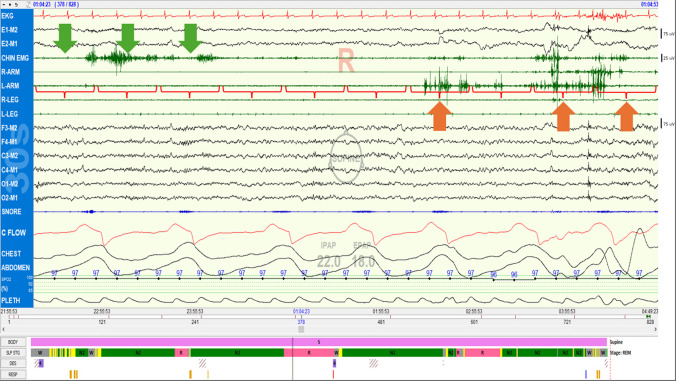
Example of REM sleep without atonia (RSWA) scoring using chin and flexor digitorum superficialis EMG

RSWA was scored when any of the following criteria were met as illustrated in Fig. 1:

1. Excessive sustained muscle activity (tonic activity): An epoch of REM sleep with at least 50% of the duration having a chin EMG amplitude at least 2 times the REM atonia level (or lowest amplitude in NREM sleep if no REM atonia is present). Each segment must be > 5 s.
2. Excessive transient muscle activity (phasic activity): In a 30-s REM epoch divided into 10 sequential 3-s mini-epochs, at least 5 (50%) of the mini-epochs contain bursts of transient muscle activity in the chin or FDS EMG. Excessive transient muscle activity bursts are 0.1–5.0 s in duration and at least 2 times the amplitude of the REM atonia level (or lowest amplitude in NREM sleep if no REM atonia is present).
3. Any EMG activity: At least 50% of 3-s mini-epochs contain any chin EMG activity with a minimum amplitude of 2 times greater than the REM atonia level (or lowest amplitude in NREM sleep if no REM atonia is present) regardless of duration (including bursts of 5–15 s) or chin or FDS EMG activity (bursts 0.1–5 s and at least 2 times as high in amplitude as the REM atonia level (or lowest amplitude in NREM sleep if no REM atonia is present).

### RSWA scoring quality assessment

RSWA was manually scored by sleep neurologists using standardized AASM criteria applied to chin and bilateral FDS EMG [17, 18]. Formal inter-reader and intra-reader reliability testing was not performed because this study used an existing clinical database, and multiple scorers did not independently score the same PSGs or REM epochs. As a quality assessment, we compared the average RSWA% across scorers and found no significant difference between sleep neurologists, suggesting no systematic difference in average scoring tendency. However, this analysis does not represent formal inter-reader or intra-reader reliability testing.

### Statistical analysis

The mean percentage of REM epochs meeting criteria for RSWA and the percentage of studies with > 27% of REM sleep epochs meeting RSWA criteria (thus meeting criteria for RBD [11, 19]) were compared between AD and non-AD groups. We performed regression analysis to measure the association between use of SSRIs, SNRIs, TCAs, and RSWA%. We employed the Ordinary Least Squares (OLS) method using Python (v3.11.15) and Statsmodels library (v0.14.4); statistical tests were computed using Scipy library (v1.11.1) to analyze the impact of AD use on RSWA variables. The model was adjusted for age, BMI, gender, and race. All *p*-values were adjusted using the Hommel method for multiple comparisons [20].

## Results

### Sample characteristics

Characteristics of the sample of 1474 patients: 624 (42.3%) were taking ADs and 850 (57.7%) were non-users (Table 1). Mean age was 53.0 ± 16.4 years (yr), 53.1% males, 73.1% White. The AD group was predominately female (57.7% vs 38.9%, *p* < 0.001), younger (52.0 ± 15.9 vs 53.8 ± 16.7 years), and White (78.9% vs 68.9%, *p* = 0.005).

**Table 1:** Sample characteristics

	Overall (*N* = 1474)	Non-users (*N* = 850)	Antidepressant use (*N* = 624)	*P*-value
Age	53.0 (16.4)	53.8 (16.7)	52.0 (15.9)	0.037
BMI	32.4 (8.4)	32.0 (8.5)	33.0 (8.3)	0.037
Gender				< 0.001
Female	691 (46.9)	331 (38.9)	360 (57.7)	
Male	783 (53.1)	519 (61.1)	264 (42.3)	
Race				0.005
White	1073 (73.1)	583 (68.9)	490 (78.9)	
Black	263 (17.9)	176 (20.8)	87 (14.0)	
Other	55 (3.7)	37 (4.4)	18 (2.9)	
Multiracial	47 (3.2)	29 (3.4)	18 (2.9)	
Asians	21 (1.4)	15 (1.8)	6 (1.0)	
American Indian	8 (0.5)	6 (0.7)	2 (0.3)	
RSWA%	11.9 (17.8)	9.7 (16.7)	15.0 (18.8)	< 0.001
RBD criteria	215 (14.6)	97 (11.4)	118 (18.9)	< 0.001

Values represent demographic and polysomnographic characteristics for the overall sample, antidepressant (AD) users, and non-users. Continuous variables are presented as mean (SD), and categorical variables as number (%). Group comparisons were performed using two-sample *t*-tests for continuous variables and chi-square tests for categorical variables. *p*-values were adjusted using the Hommel method to account for multiple comparisons

Table 2 summarizes AD use by drug type. The majority (*n* = 580, 92.9%) were on AD monotherapy including SSRIs (*n* = 377, 60.4%), SNRIs (*n* = 171, 27.4%), and TCAs (*n* = 32, 5.1%). Combination therapy included SSRI + SNRI (*n* = 14, 2.3%), SSRI + TCA (*n* = 19, 3.1%), and SNRI + TCA (*n* = 9, 1.4%).

**Table 2:** RSWA percentage by group

Medication category	*N* (%)	*N* (%) Meeting rbd criteria	Mean RSWA%	Antidepressant use* (conf. interval)	*P*-value**
SSRI only	377 (25.6)	58 (15.6)	13.77 ± 18.32	3.39 (1.37, 5.41)	**0.009**
SNRI only	171 (11.6)	43 (25.1)	17.43 ± 19.21	6.66 (3.92, 9.40)	** < 0.001**
TCA only	32 (2.17)	2 (6.25)	8.00 ± 12.51	− 3.57 (− 9.59, 2.46)	0.278
SSRI + SNRI	14 (0.95)	5 (35.7)	20.86 ± 20.97	10.44 (1.02, 19.86)	0.089
SSRI + TCA	19 (1.29)	6 (31.6)	17.45 ± 21.31	4.37 (− 3.45, 12.18)	0.278
SNRI + TCA	9 (0.61)	3 (33.3)	24.34 ± 28.28	12.81 (1.53, 24.09)	0.078

^*^Linear regression coefficient for medication use when predicting mean % (SD) of REM epochs. The medication coefficient was adjusted for age, BMI, gender, and race

^**^Compared with non-users SSRI, SNRI, or TCAs (*N* = 850, % (SD) of REM Epochs with RSWA 9.71 (16.70)). *p*-values have been adjusted using the Hommel method to account for multiple comparisons

### Association between RSWA variables and AD use

Mean RSWA% was significantly higher among AD users than non-users (15.0% ± 18.8 vs 9.7% ± 16.7, *p* < 0.001). Studies meeting the > 27% RSWA cutoff for RBD were significantly greater among AD users than non-users (18.9% vs 11.4%, *p* < 0.001). RSWA data are shown in Table 2 overall and by AD group. Compared to non-users and after adjusting for age, BMI, gender, and race, monotherapy with SSRIs was associated with a 3.39% (95% CI = 1.37% to 5.41%, *p* = 0.005) increase in RSWA%, SNRIs a 6.66% (95% CI = 3.92% to 9.40%, *p* < 0.001) increase, and TCAs a 3.57% decrease (95% CI = − 9.59 to 2.46, *p* = 0.246).

Combined AD use demonstrated varying degrees of association with RSWA. After adjustment, use of SSRI + SNRI showed an increase in RSWA% of 10.44% (95% CI = 1.02% to 19.86%, *p* = 0.089) relative to no drug use. Those using SNRI + TCA exhibited an even greater increase of 12.81% (95% CI = 1.53% to 24.09%, *p* = 0.078). The combination of SSRI + TCA was associated with a 4.37% increase (95% CI = −3.45% to 12.18%, *p* = 0.278).

Electrodiagnostic criteria for RBD was met in 58 (15.6%) SSRI users, 43 (25.1%) SNRI users, and 2 (6.25%) TCA users. RBD criteria were also met by 5 (35.7%) SSRI + SNRI users, 6 (31.6%) SSRI + TCA users, and 3 (33.3%) SNRI + TCA users.

## Discussion

We leveraged the Cleveland Clinic STARLIT Registry to examine 1474 vPSGs that underwent manual RSWA scoring because of suspected parasomnia. RSWA was scored using chin and bilateral FDS EMG according to AASM criteria [17, 18] to study the association of AD use with RSWA% and RBD diagnosis. We found (1) SSRI and SNRI monotherapy use was associated with a higher percentage of RSWA epoch, and PSGs meeting RBD diagnosis versus non-users; (2) the highest RSWA% estimates among combination groups were observed with SSRI + SNRI, although combination subgroup findings should be interpreted cautiously given small sample sizes.; (3) no association was found between TCA use alone and RSWA%; and (4) the proportion of patients meeting criteria for RBD was higher among those taking ADs compared with non-users.

### Prior studies quantifying RSWA in people taking antidepressants were small and did not record upper extremity EMG

The largest study investigating AD effects on RSWA% used qualitative visual inspection of EMG in REM sleep (identifying only the presence/absence of RSWA) in 10,746 PSGs, and excluded TCAs [9]. They found any RSWA in at least one epoch of REM sleep in 12.2% of PSGs of patients using ADs compared with 2.1% in the entire cohort of AD users and non-users. As expected given the younger cohort, the prevalence of RBD, defined by the presence of RSWA with a history DEB (by chart review, vPSG, or pre-PSG questionnaire), was lower among AD users (0.48%) than in the overall cohort (1.0%; *p*= 0.005), suggesting that while SSRIs and SNRIs are strongly associated with RSWA, but their link to clinical RBD may be less pronounced [9].

Only three prior investigations have quantified RSWA by AD use, all with smaller sample sizes and different recording and scoring criteria. All used the Lapierre-Montplaisir method defining (1) tonic RSWA when chin EMG activity amplitude ≥ 2 times the background or > 10 µV is present for ≥ 50% of the epoch and atonia when it is present for < 50 and (2) phasic chin EMG as the percentage of 2- or 3-s mini epoch according to which adaptation used [12, 21] containing EMG bursts lasting 0.1–10 s with amplitude > 4 times the background; leg movements during REM are also included from bilateral AT EMG as events lasting 0.1–10 s with amplitude > 4 times the background [22]. In addition to the different adaptations of the Montplaisir method, two studies only included serotonergic AD such as sertraline [10, 21], whereas the third included ADs without specifying the class. These methodological differences likely contribute to the variability in reported RSWA percentages [12].

In the first, elevated tonic submental EMG was observed in 9.54% ± 9.06 of REM epochs in 15 AD users versus 2.36% ± 3.88 in 15 matched controls (*p* = 0.02) [10]. In the second, combined submental and/or AT RSWA activity averaged 36.9% ± 21.3 in 30 AD psychiatric patients compared to 15.9% ± 8.4 in 15 non-user psychiatric patients (*p* < 0.01) [12]. The third investigated the effect of adding sertraline in 31 patients with depression and found RSWA increased from day 1 to day 56 for both submental (3.4 ± 1.9% vs. 11.4 ± 4.2%; *p* < 0.001) and AT (6.2 ± 2.1% vs 15.1 ± 6.6%; *p*< 0.001) EMG [21]. No clinical RBD behaviors accompanied RSWA on video-PSG [21].

### Recent studies confirming submentalis and flexor digitorum superficialis best diagnostic marker for isolated RBD

We recorded and scored RSWA using the FDS EMG rather than AT because the AT leads are prone to motion and electrode artifact which can lead to false-positive findings [23]. Recent studies evaluating diagnostic accuracy confirm that manual scoring of phasic/tonic submental + phasic FDS has the best performance for the diagnosis of RBD [23]. Frauscher et al. systematically recorded EMG in 11muscles of 30 patients with RBD, 15 with PD, and 30 matched controls, and reported the discriminative power for identifying RSWA was higher in the upper limb (100% specificity, area under the curve (AUC) 0.987–9.997) than in lower limb muscles (100% specificity, AUC 0.813–0.852) [11]. Furthermore, a 2023 meta-analysis (14 studies, 434 subjects) found that AASM RSWA scoring methods demonstrated the highest diagnostic accuracy for RBD at 92.2%, with a sensitivity of 92% and specificity of 99% while the Lapierre-Montplaisir RSWA scoring system showed a lower accuracy of 84.8% [24].

### Possible mechanisms for association between antidepressant use and RSWA

REM sleep atonia in humans is regulated by the glutamate-releasing neurons in the pontine sublaterodorsal nucleus which activate gamma-aminobutyric acid and glycine releasing premotor neurons in the gigantocellular and magnocellular nuclei in the ventral medulla which inhibit spinal motoneurons thereby causing motor paralysis in REM sleep. The observed association between SSRIs/SNRIs and RSWA may be due to activation of dorsal raphe serotonergic and locus coeruleus noradrenergic brainstem nuclei during REM sleep inhibiting normal REM atonia [25, 26]. This aligns with the hypothesis that serotonergic ADs primarily unmask subclinical RBD rather than cause it de novo, as patients with AD-associated RBD demonstrate neurodegenerative markers such as olfactory impairment, color vision deficits, and autonomic dysfunction that cannot be explained by AD use alone [16].

### Limitations

These findings may be relevant to RBD research because AD use is common among patients referred for sleep evaluation; however, the clinical selection of this cohort limits generalizability to broader populations.

Strengths of our work include robust RSWA scoring methodology performed by four experienced sleep neurologists using current AASM criteria applied to a large clinical dataset. However, our study has limitations inherent to its cross-sectional design. First, this cohort was derived from a clinical database of vPSGs selected for manual RSWA scoring; the sample was enriched for patients with suspected parasomnia. This selection process likely increases the overall percentage of RSWA compared with an unselected sleep laboratory population. Therefore, the RSWA percentages reported in this study should not be interpreted as prevalence estimates for all patients taking ADs or for all patients undergoing vPSG. Rather, these findings apply specifically to patients who met clinical criteria for RSWA scoring at our center and should be interpreted within that context. Second, causality between AD use and RSWA/RBD diagnosis cannot be inferred. Third, we could not assess the impact of potential confounders such as use of melatonin and other therapies for RBD or medical comorbidities. Fourth, because the annotation software does not retain information distinguishing tonic from phasic EMG activity during label processing, we were unable to separately quantify tonic and phasic RSWA. Lastly, because our sample included a higher proportion of White participants, and the AD group had a higher proportion of females and was slightly younger than the non-user group, our findings may not be generalizable to community or more diverse populations. Importantly, although our methodology represents one of the most rigorous approaches to RSWA quantification to date, the process is highly labor intensive limiting its scalability across centers.

## Conclusions

In this clinically selected cohort of patients who underwent manual RSWA scoring for suspected parasomnia, parasomnia type symptoms or behaviors, or concern for increased REM EMG tone, we report a higher RSWA% among patients taking ADs, including combination therapy. Because the cohort was enriched for patients with clinical concern for parasomnia or increased REM EMG activity, the RSWA percentages reported here should not be interpreted as prevalence estimates for all patients taking ADs or for unselected sleep laboratory populations. Future studies in broader, unselected cohorts are warranted.

## Supplementary Information

Below is the link to the electronic supplementary material.

ESM 1 (DOCX 15.0 KB)

## Data Availability

The data that supports the findings of this study are available from the corresponding author upon reasonable request.

## Abbreviations

AUC: Area under the curve
AASM: American Academy of Sleep Medicine
AD: Antidepressant
AT: Anterior tibialis
DEB: Dream enactment behaviors
DLB: Dementia with Lewy bodies
DOR: Diagnostic odds ratio
EHR: Electronic health record
EMG: Electromyography
FDS: Flexor digitorum superficialis
MSA: Multiple system atrophy
PD: Parkinson’s disease
PSG: Polysomnography
RBD: REM sleep behavior disorder
RSWA: Rapid eye movement sleep without atonia
SNRI: Serotonin norepinephrine reuptake inhibitor
SSRI: Selective serotonin reuptake inhibitor
TCA: Tricyclic antidepressant
vPSG: Video polysomnography
