# Supplementary material for: Antidepressant medications have differential effects on REM sleep without atonia quantified by chin and upper extremity EMG
Source: J Clin Sleep Med. 2026 Jul 9;22(1):109. doi: 10.1007/s44470-026-00127-2 (PMC13350564; doi:10.1007/s44470-026-00127-2)
Supplement: Supplementary file 1 — (DOCX 15.0 KB) [file 44470_2026_127_MOESM1_ESM.docx]

| **Class** | **Generic Name** | **Brand Names** |
| --- | --- | --- |
| SSRI | Citalopram | Celexa |
|  | Escitalopram | Lexapro |
|  | Fluoxetine | Prozac |
|  | Fluvoxamine | Luvox |
|  | Paroxetine | Paxil  Paxil CR |
|  | Sertraline | Zoloft |
| SNRI | Duloxetine | Cymbalta |
| SNRI | Desvenlafaxine | Pristiq |
| SNRI | Levomilnacipran | Fetzima |
| SNRI | Venlafaxine | Effexor  Effexor XR |
| TCA | Amitriptyline | Elavil |
| TCA | Clomipramine | Anafranil |
| TCA | Doxepin | Silenor  Sinequan |
| TCA | Imipramine | Tofranil |
| TCA | Nortriptyline | Pamelor |

Supplemental Table. Antidepressant Medications Included in the SSRI, SNRI, and TCA Categories
